# Binary colloidal crystals (BCCs) as a feeder-free system to generate human induced pluripotent stem cells (hiPSCs)

**DOI:** 10.1038/srep36845

**Published:** 2016-11-11

**Authors:** Peng-Yuan Wang, Sandy Shen-Chi Hung, Helmut Thissen, Peter Kingshott, Raymond Ching-Bong Wong

**Affiliations:** 1 Department of Chemistry and Biotechnology, Faculty of Science, Engineering and Technology, Swinburne University of Technology, Hawthorn, Victoria 3122, Australia; 2 Graduate Institute of Nanomedicine and Medical Engineering, College of Biomedical Engineering, Taipei Medical University, Taipei 110, Taiwan; 3 Department of Anatomy and Neuroscience, Florey Neuroscience and Mental Health Institute, The University of Melbourne, Victoria 3000, Australia; 4 CSIRO Manufacturing, Bayview Avenue, Clayton, 3168 Victoria, Australia; 5 Centre for Eye Research Australia, Royal Victorian Eye and Ear Hospital; Ophthalmology, Department of Surgery, University of Melbourne, Victoria 3002, Australia

## Abstract

Human induced pluripotent stem cells (hiPSCs) are capable of differentiating into any cell type and provide significant advances to cell therapy and regenerative medicine. However, the current protocol for hiPSC generation is relatively inefficient and often results in many partially reprogrammed colonies, which increases the cost and reduces the applicability of hiPSCs. Biophysical stimulation, in particular from tuning cell-surface interactions, can trigger specific cellular responses that could in turn promote the reprogramming process. In this study, human fibroblasts were reprogrammed into hiPSCs using a feeder-free system and episomal vectors using novel substrates based on binary colloidal crystals (BCCs). BCCs are made from two different spherical particle materials (Si and PMMA) ranging in size from nanometers to micrometers that self-assemble into hexagonal close-packed arrays. Our results show that the BCCs, particularly those made from a crystal of 2 μm Si and 0.11 μm PMMA particles (2SiPM) facilitate the reprogramming process and increase the proportion of fully reprogrammed hiPSC colonies, even without a vitronectin coating. Subsequent isolation of clonal hiPSC lines demonstrates that they express pluripotent markers (OCT4 and TRA-1-60). This proof-of-concept study demonstrates that cell reprogramming can be improved on substrates where surface properties are tailored to the application.

Cell reprogramming of somatic cells into human induced pluripotent stem cells (hiPSCs) can potentially provide tremendous advancements to cell biology in a wide range of applications such as drug screening, disease modelling, blood transfusion and regenerative medicine^1^. The generation of hiPSCs was first reported in 2007^2^,^3^, a year after the success of mouse iPSCs^4^, and was determined by the exogenous expression of four transcription factors including OSKM (Oct3/4, Sox2, Klf4, and cMyc)^2^ or OSNL (Oct4, Sox2, Nanog, and Lin28)^3^. To date, a range of reprogramming protocols utilising different vectors, cocktails (transcription factors, chemical compounds, and miRNAs) and cell types (fibroblasts, renal epithelial cells, and epidermal keratinocytes) have been described for hiPSCs generation^5^.

However, several challenges remain with cellular reprogramming using current technologies, including issues with genomic insertion, tumorgenicity, low efficiency, and incomplete reprogramming^5^. Improvements to the reprogramming techniques have been described, such as the use of non-integrative vectors like episomal vectors^6^ or Sendai viruses^7^, and elimination of oncogenic reprogramming factors such as cMyc to address tumorigenicity issues^8^. In addition, many studies have focused on improving the efficiency of cell reprogramming using various chemical or biological strategies^9^. Furthermore, the development of feeder-free reprogramming cell culture conditions using the chemically defined E7 Medium combined with vitronectin (VN)-coated surfaces have greatly improved the feasibility of reprogramming and eliminated variations associated with feeder cells^10^.

The mechanisms underlying reprogramming are the subject of intense research. Transcriptome analysis revealed that reprogramming consists of multiple phases marked by distinct changes in gene expression and epigenetic states^11^. It is apparent that the vast majority of cells can initiate reprogramming, but many of them fail in the intermediate phases and are not fully reprogrammed into iPSCs, resulting in low reprogramming efficiency^12^. From an engineering perspective, low reprogramming efficiency will result in increased cost and time, which are critical issues in clinical applications. From a biology perspective, partially reprogrammed cells often form colonies that do not resemble ESC-like morphologies and cannot be used in further applications such as drug screening and cell therapy. In addition, manual iPSC colony isolation is a time-consuming process and the colony selection process by morphology requires highly specialised knowledge. Elimination of partially reprogrammed colonies in culture would simplify the process of colony isolation and improve the quality of downstream applications.

Apart from biochemical methods, biophysical strategies have recently been proposed to improve the efficiency of cell and lineage reprogramming^13^,^14^,^15^. This strategy utilises cell-surface interactions, a well-defined and stable method, to modulate the cellular response. Downing *et al*. first reported that the surface topography of a cell culture substrate, in the form of parallel microgrooves or nanofibers, can replace the effects of small-molecule epigenetic modifiers and significantly improve reprogramming efficiency of mouse fibroblasts into iPSCs^13^. They postulate that a mechano-modulation effect of the substrate topography can modulate the epigenetic state of the cells. Specifically, patterned surfaces lead to increased histone H3 acetylation and methylation. They also showed that patterned surfaces promoted a mesenchymal-to-epithelial transition (MET) in mouse fibroblasts. Recently, similar strategies have been used for direct reprogramming of fibroblasts into neurons^14^, and cardiac progenitor cells into cardiomyocytes^15^ using microgroove-based surface topographies. These pioneer studies all show that cell morphology and cytoskeleton change on the topographical patterns and subsequently improve cell reprogramming. Therefore, we hypothesize that modulation of the cytoskeleton of cultured cells using specific topography/chemistry can improve the reprogramming process.

We have recently developed a new type of surface based on binary colloidal crystals (BCCs)^16^ for use in adult cell and stem cell culture^17^. The results showed that the cytoskeleton of human MSCs was changed and the cells formed clumps on BCCs after 4 day culture^17^. Although there are similar studies using particle substrates for mouse ESC culture^18^, there is so far no report using particles or similar structures for cell reprogramming. BCCs are composed of hexagonal close-packed structures made of mixtures of spherical nano- and micro-particles (from 100 nm to 5 μm with a size ratio (small-to-large) <0.2), which spontaneously form by self-assembly onto flat surfaces from dilute suspensions. BCCs are easy to fabricate, have tuneable surface properties (i.e. roughness and wettability using different particles sizes and material chemistry), and can be prepared over quite large areas (i.e. surface coverage = 7 cm^2^) within a few hours, which is difficult to accomplish using other micro-/nanofabrication technologies. The symmetry of the surface topography can be modulated using different ratios of colloids in suspension, and the topography can be varied from random to highly ordered. All these advantages support the feasibility of using BCCs as the next generation cell culture tools, and potentially as a surface modification strategy for applications in regenerative medicine.

In this study, the applicability of BCCs as a substrate for reprogramming human fibroblasts to hiPSCs using a non-integrative, feeder-free system was tested. Two BCCs (2 μm silica (Si)/0.1 μm poly(methyl methacrylate) (PMMA) = 2SiPM, and 5 μm Si/0.4 μm PMMA = 5SiPM) with a high quality ordered surface symmetry and defined topography were selected as substrates. We compared the reprogramming processes that occur on BCCs with flat glass and non-tissue culture treated polystyrene (PS) controls in the presence or absence of a vitronectin (VN) coating. Surface topography and surface chemistry-induced changes in terms of cell morphology, growth, and reprogramming of human fibroblasts were investigated. The aim of this study was to improve cellular reprogramming processes by using complex surface topographies and chemistries that can be easily manufactured.

## Materials and Methods

### BCC fabrication and characterization

Monolayer binary colloidal crystals (BCCs) were fabricated according to our previous protocol using evaporation induced confined area assembly (EICAA)^16^,^17^. Briefly, two BCCs, 2SiPM (2 μm silica particles (Si) and 0.11 μm PMMA) and 5SiPM (5 μm Si particles and 0.4 μm PMMA) were selected for cell culture. BCCs were fabricated by mixing two colloids in Milli-Q water prior to depositing them in a confined area (i.e. a 3-cm-diameter rubber ring) on PS-coated glass slides. A 5% (w/v) PS solution in toluene was spin-coated at 800 rpm for 1 min. The volumes of the two colloidal solutions were calculated based on the concentration of large Si particles needed to form a monolayer inside the confined area. In this study, approx. 34 million 5 μm Si particles and 5,300 million 400 nm PMMA particles were needed, and approx. 210 million 2 μm Si particles and 71,000 million 110 nm PMMA were needed to form BCCs inside the 3-cm-diameter O-ring (7 cm^2^). After water evaporation and BCC formation surfaces were heated to 200 °C for 1 min to stabilise the particle layers where the PMMA is partially melted and acts as a glue. BCCs were then sterilised with a UV/Ozone (ProCleaner™ Plus, BioForce Nanosciences, Inc.) for 30 min. Commercially available polystyrene plate (PS; Falcon) was used as a positive control and flat glass slides were used as a topographical control due to the chemical similarity between glass and Si particles.

The surface structure of the BCCs was analysed using field emission scanning electron microscopy (FE-SEM; ZEISS SUPRA 40 VP, Carl Zeiss, Germany) at 20 keV after deposition of a 10–15 nm gold coating. Fast Fourier transformations (FFTs) was used to confirm the crystallization of BCCs using ImageJ software. The surface roughness of the substrates was analysed using atomic force microscopy (AFM) with a scan rate of 0.5 Hz (AFM, Dimension iCon, Bruker Corp., Billerica, MA, USA). A surface area of 10 × 10 μm and 25 × 25 μm on 2SiPM and 5SiPM was scanned, respectively. Surface roughnesses at a total of six spots on three different samples was averaged (*n* = 6). The surface wettability of the BCC monolayers was determined using sessile drop water contact angle (WCA) measurements (KSV instruments Ltd, Finland) using 5 μL Milli-Q water. A total of five spots on two different samples was analysed (*n* = 10).

The surface chemistry of various surfaces was determined using X-ray photoelectron spectroscopy (XPS, AXIS Nova, Kratos Analytical Ltd., Manchester, UK). A monochromated Al_kα_ X-ray source operating at a power of 150 W was applied. Surfaces were analysed prior to and after a vitronectin coating. After 1 h coating, surfaces were rinsed twice with PBS and dried by N_2_. Survey and high resolution spectra were acquired at 160 eV and 20 eV pass energies, respectively. Six spots on each surface with an elliptical area of approx. 300 × 700 μm^2^ were analysed (*n* = 6). Data analysis and quantification were performed using CasaXPS processing software version 2.3.16 (Casa Software Ltd. Teignmouth, UK). All elements in the survey spectra were quantified and presented as percentage of each atom. High resolution C 1s, N 1s, and O 1s spectra were also analysed to further confirm the structure of the vitronectin coating.

### Culture and characterization of fibroblasts on BCCs

All cell culture experiments involving human cell lines were approved by the Human Research Ethics committees of the Royal Victorian Eye and Ear Hospital (11/1031H, 13/1151H-004) and the University of Melbourne (0605017, 0829937) and carried out in accordance with the approved guidelines. Informed consent was obtained from all subjects. BJ human foreskin fibroblasts were cultured in DMEM medium supplemented with 10% fetal calf serum, 1x L-glutamine (2 mM), 0.1 mM non-essential amino acids and 0.5x (50 U/mL) penicillin/streptomycin (all from Invitrogen).

Cell adhesion, morphology and proliferation of BJ human fibroblasts was analysed on each surface after 1 and 3 days using ImageJ software according to a previous study^19^. After 3 days, fibroblasts were confluent on the PS control with cell-cell contacts influencing the cell morphology and quantification. Therefore, the size and aspect ratio (cell length/width) was characterized at day 1 only. Briefly, at each time point, cells were fixed with 4% (w/v) paraformaldehyde, permeabilized with 0.1% (v/v) Triton-X100 and surfaces blocked with 1% (w/v) bovine serum albumin. The samples were then immunostained with F-actin, followed by phalloidin–TRITC (500 nM in PBS) and DAPI counterstain (100 nM in PBS). Fluorescent images were captured using an inverted Epi-fluorescence microscopy (Eclipse Ti-E, Nikon Instruments Inc., Japan). Cell density was quantified using DAPI stained images (*n* = 10 images). The cell size and aspect ratio (length/width) were quantified using F-actin stained images (*n* > 100 cells).

### Reprogramming to hiPSCs

**Figure 1:**
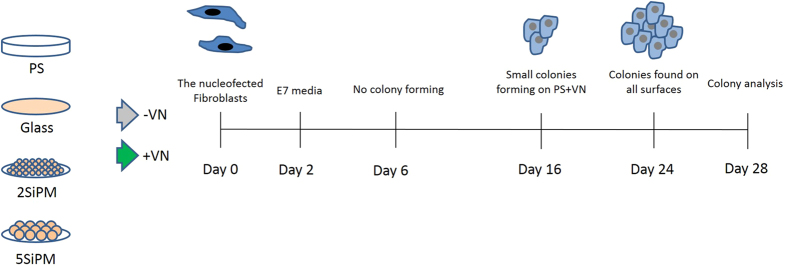
An overview of cell reprogramming on binary colloidal crystals (BCCs). Two selected BCCs (2SiPM and 5SiPM), control (glass), and a gold standard surface (PS) were either without any coating or coated with vitronectin (VN). Episomal vectors expressing OCT4, SOX2, KLF4, L-MYC, LIN28 and shRNA against p53 were transfected into human fibroblasts. After 2 day culture, medium was switched to E7 medium for cell reprogramming. Cell morphology was monitored every day and colonies were characterized at day 28.

hiPSCs were generated using BJ human fibroblasts with episomal vectors in a feeder-free system as depicted in Fig. 1 6,20. Briefly, 50,000 fibroblasts were nucleofected with episomal vectors expressing OCT4, SOX2, KLF4, L-MYC, LIN28 and shRNA against p53 (plasmids were gifts from Prof. Shinya Yamanaka; gene plasmid #27077, #27078, #27080). The nucleofected fibroblasts were plated on the specified surface coating with or without vitronectin (VN) in fibroblast media (Day 0). VN (10 μg/mL, Stem Cell Technologies) was coated on the various surfaces at room temperature for 1 hour prior to cell seeding. From day 2 onwards, the fibroblast medium are switched to E7 medium (Stem Cell Technologies) for cell reprogramming. The medium was changed every day.

On day 28, the reprogrammed cultures were immunostained using the alkaline phosphatase (AP) detection kit following the manufacturer’s protocol (cat#86R-1KT, Sigma). Quantification of fully reprogrammed hiPSC colonies was performed by counting the colonies that resemble hESC morphology and are positive for AP expression. This quantification approach has been shown to yield similar numbers of TRA-1-60 positive colonies, demonstrating this as a valid way to identify fully reprogramed hiPSC colonies (Supplementary Fig. 1)^20^. Subsequently, a clonal hiPSC cell line was isolated from reprogramming from the 2SiPM or 5SiPM substrates. The isolated clonal hiPSCs were maintained on mitotically inactivated mouse embryonic fibroblasts feeders in the presence of DMEM/F-12 medium containing 1x GlutaMAX, 20% knockout serum replacement, 10 ng/ml basic fibroblast growth factor, 0.1 mM non-essential amino acids, 100 μM β-mercaptoethanol and 0.5x (50 U/mL) penicillin/streptomycin (all from Invitrogen). Further characterization of hiPSCs was performed to assess the expression of pluripotent markers OCT4 and TRA-1-60, as described previously^21^. Briefly, immunocytochemistry was performed with antibodies against OCT4 (#sc-5279, Santa Cruz Biotechnology) or TRA-1-60 (#AB16288, Abcam), followed by the appropriate Alexa-fluor 488 and DAPI counterstaining (Invitrogen). The samples were imaged using a Nikon Eclipse TE2000 inverted microscope.

### Statistical analysis

Statistical analysis was performed using the GraphPad 3.0 program (GraphPad Software, La Jolla, CA). Data handling was performed in Microsoft Excel or OriginPro. The statistical analysis between each group was determined with one-way ANOVA and Student–Newman–Keuls multiple comparisons test. *p* < 0.05 was use to establish statistical significance.

## Results

### Surface characterization of BCCs

**Figure 2:**
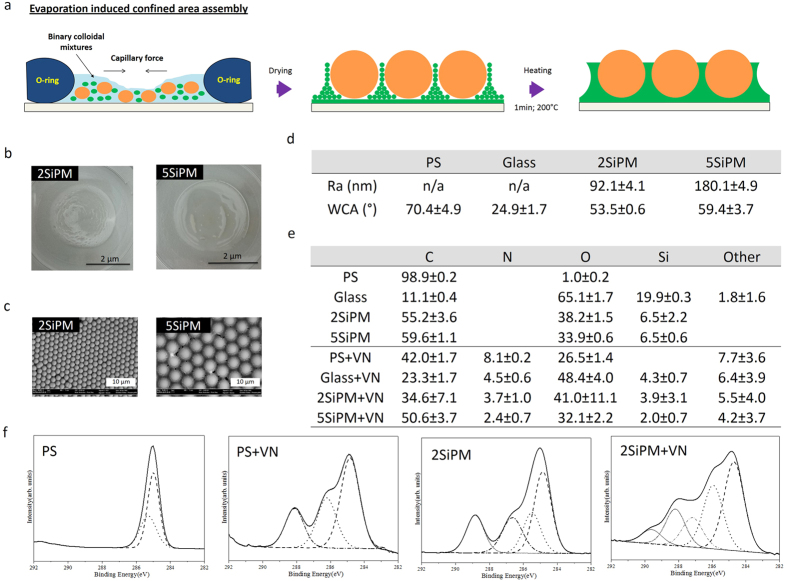
Surface characterization of BCCs. (**a**) Schematic illustration of binary colloidal crystal (BCC) fabrication. (**b**) Photos of two selected BCCs, i.e. 2SiPM and 5SiPM. While the transparent part on the surface indicates a monolayer, the white part indicates a multilayer. Both areas have a hcp structure. (**c**) Scanning electron microscopy (SEM) images of the two BCCs. (**d**) Surface roughness (Ra) and water contact angle (WCA) measurements of the two BCCs. Values = mean ± SD (*n* = 6). (**e**) X-ray photoelectron spectroscopy (XPS) elemental analysis of surfaces with or without a vitronectin (VN) coating determined from survey spectra. Values = mean ± SD (*n* = 6). Some data in (**d**,**e**) was published previously^17^. C = carbon; N = nitrogen; O = oxygen; Si = silicon; other = other elements including phosphorus, sodium, chloride, and zinc (detail also see Supporting Information Table S1). (**f**) High resolution C 1s XPS spectra of PS and 2SiPM with or without VN coating. The spectra demonstrated a different vitronectin structure on various surfaces (more detail also see Supplementary Fig. 2).

A brief schematic illustration was depicted in Fig. 2a. The two selected BCCs (2SiPM and 5SiPM) came from our library consisting of over 100 combinations, and is based on those crystals showing the highest frequencies of hexagonal close-packed (hcp) surface topographies^17^,^22^. The surface properties of the BCCs and controls (i.e. glass, and PS) were characterized using scanning electron microscopy (SEM), atomic force microscopy (AFM), water contact angle (WCA) measurements, and X-ray photoelectron spectroscopy (XPS) (Fig. 2b–f).

The surface topography of BCCs was highly ordered locally they were not perfectly uniform across the surface (a combination of monolayer and multilayer areas was observed; Fig. 2b). Although the nature of self-assembly process cannot provide a perfect monolayer, the top surface structure still provides hcp surface structures. From the SEM and AFM images (Fig. 2b,c), BCCs were composed of large Si particles arranged in a hcp structure surrounded by small PMMA particles, which act as a glue to stabilize the structure after partial melting. The surface roughness of the 5SiPM samples (180.1 ± 4.9 nm) was twice that of the 2SiPM samples (92.1 ± 4.1 nm) (Fig. 2d). However, the surface wettability as determined from sessile water contact angles (2SiPM: 53.5° ± 0.6°; 5SiPM: 59.4° ± 3.7°, Fig. 2d) and average elemental composition (55–59% carbon, 38–34% oxygen, and 6.5% silicon) was similar for the two BCC substrates (Fig. 2e). In comparison, the surface properties of flat surface controls were substantially different. The PS surface was essentially composed of carbon (~99%) while glass had a high oxygen (~65%) and low carbon content (~11%) with about 20% silicon. While PS was more hydrophobic (WCA ~70.4° ± 4.9°), glass was more hydrophilic (WCA ~34.8° ± 1.6°) compared to the BCCs (Fig. 2e).

XPS analysis was performed on all surfaces before and after adsorption of vitronectin (VN), an extracellular matrix protein used in feeder-free reprogramming methods. After the VN coating, substantial amounts of nitrogen and smaller amounts of phosphorus, sodium, chloride, and zinc were detected on all surfaces to varying degrees (Fig. 2e; and Supplementary Table 1). The Si signal indicated a very thin VN layer (<10 nm) coated on the surfaces. It is apparent that the surface properties influence the quantity of VN that adsorbs to each substrate. The nitrogen content is a measure of the level of protein adsorption since it arises from the peptide structure of the protein^16^. In this respect, the quantity of adsorbed VN follows the trend: PS (8.16 ± 0.29%N) > glass (4.53 ± 0.65%N) > 2SiPM (3.75 ± 1.09%N) > 5SiPM (2.46 ± 0.71%N). It is likely that not only the quantity is variable between these surfaces but also the molecular conformation of VN according to high resolution spectra (Fig. 2f). The results showed that the surface chemistry of each surface affected the vitronectin coating. For example, the C1s spectra for PS+VN has only three chemical structures while for 2SiPM+VN there are five chemical structures (two extra peaks at 287 and 290 eV; Fig. 2f, and Supplementary Fig. 2). The N1s spectra for PS+VN and glass+VN has only one peak (binding energy at 400 eV), while there were three N peaks detected on the 2SiPM+VN surface (Supplementary Fig. 2) suggesting that the VN structure was influenced by the different surface properties.

### Human fibroblasts cultured on BCCs

**Figure 3:**
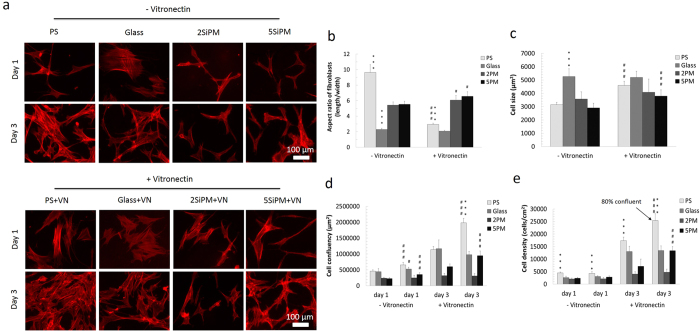
Characterization of human fibroblasts cultured on BCCs. (**a**) F-actin staining of fibroblasts on the two BCCs and controls with or without a vitronectin (VN) coating. Nucleus staining images are in the Supporting Information Fig. S2. (**b**) Aspect ratio of cells (cell length/width) after 1 day culture. (**c**) Cell size after 1 day culture. Quantification of: (**d**) cell density, and (**e**) cell confluency on the different surfaces after 3 day culture. Value = mean ± SD (*n* = 10). *vs. other surfaces with the same surface treatment at the same day, and ^#^vs. the same surface without a vitronectin (VN) coating at the same day. One, two, and three symbols indicate *p* < 0.05, 0.01, and 0.001, respectively.

The adhesion, morphology, and growth of human fibroblasts on different surfaces were studied at day 1 and day 3 after seeding (Fig. 3). Cytoskeleton (F-actin) staining showed different cell morphologies on the different surfaces (Fig. 3a, and 4x images in Supplementary Fig. 3). After day 1, cells were more elongated on PS and BCCs while those on glass were wider (triangle- or quadrangle-like shape, Fig. 3a). In the presence of VN, cells on PS+VN adapt a wide morphology while there was no obvious change in morphology with cells attached to glass+VN compared with non-VN coated glass (Fig. 3a). On the BCCs+VN substrates, cells became larger (longer and wider) compared with those on BCCs without a VN coating (Fig. 3a). At day 1, cells were less confluent allowing for the analysis of individual cells (Fig. 3b,c). The aspect ratio of cells (cell length/width) showed that the ratio of cells on PS decreased significantly due to an increase of width (Fig. 3b). The ratio of cells on BCCs was slightly increased due to an increase of both width and length, while on glass was no change. Cell size analysis showed that cells on glass were the largest, a response that was independent of the VN coating (Fig. 3c). However, adherent cells on PS and the two BCCs exhibited an elongated morphology with a smaller average cell size. Moreover, cells were larger after attachment to PS+VN and the two BCCs+VN. After 3 days culture, cells became confluent or had increased cell-cell contacts on surfaces which hindered the analysis of cell size and morphology. It was observed that fibroblast proliferation on BCCs was slower compared to PS and glass (Fig. 3d). Cell density was significantly higher on PS (~4,400 cells/cm^2^) compared to glass (~2,500 cells/cm^2^) and the two BCCs (~2,000 cells/cm^2^ on 2SiPM and 2300 cells/cm^2^ on 5SiPM) at day 1 (*p* < 0.001, Fig. 3d). After the VN coating, the cell density on glass+VN (~3,000 cells/cm^2^, *p* < 0.05 vs. glass) increased slightly, but not for the 2SiPM+VN (~2,100 cells/cm^2^), 5SiPM+VN (~2,800 cells/cm^2^), and PS+VN (~4,300 cells/cm^2^) surfaces. After 3 days, the cell density was the highest on PS+VN (~25,000 cells/cm^2^) with an ~6 fold increase, followed by PS (~17,000 cells/cm^2^ ~4 fold increase). The proliferation on the other surfaces was significantly different where cell densities after 3 days increased 5 fold on glass (~13,000 cells/cm^2^), 4.3 fold on glass +VN (~13,000 cells/cm^2^), 3 fold on 5SiPM (~7,100 cells/cm^2^), 4.7 fold on 5SiPM+VN (~13,000 cells/cm^2^), 1.9 fold on 2SiPM (~4,000 cells/cm^2^), and 2.3 fold on 2SiPM+VN (~4,700 cells/cm^2^).

Consistent with these results, after 3 days the degree of cell confluency on glass and PS was higher compared to the BCCs. In the presence of VN coatings, the cell confluency was observed to increase on all surfaces except 2SiPM. After 3 days, cells were ~80% confluent on PS+VN with the highest surface coverage (~1.98 × 10^6^ μm^2^ in a 4x image, Fig. 3e). After 3 day, the cell confluency on glass, glass+VN and 5SiPM+VN was ~50%, ~25% on 5SiPM+VN, and ~15% on 2SiPM and 2SiPM+VN. Interestingly, the VN coating had no influence on the initial cell attachment (24 h) on all surfaces except glass, but VN did affect cell proliferation after 3 days, with a surface dependent variation. Overall, the fibroblast proliferation rate was highest on PS and lowest on 2SiPM.

### Cell reprogramming on BCCs

**Figure 4:**
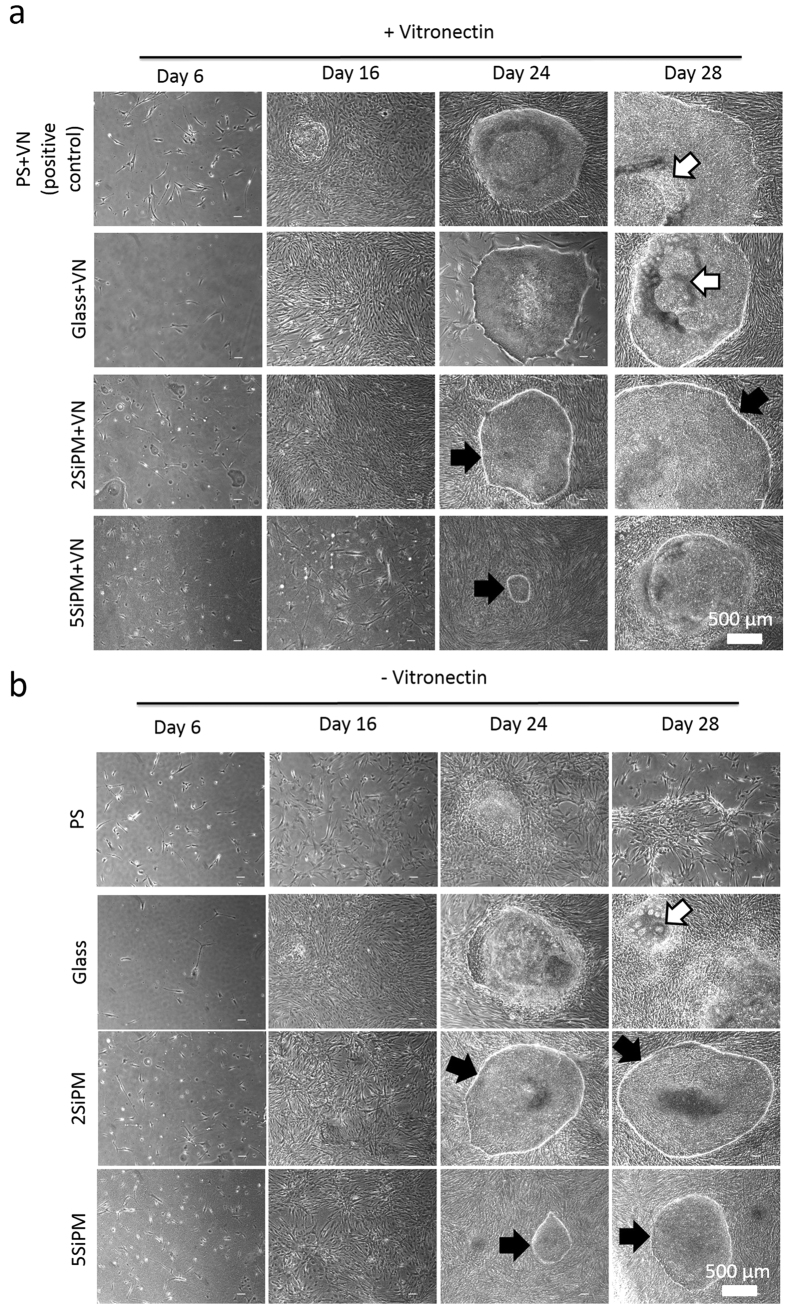
Cell morphology during reprogramming. Representative morphologies are shown at different time points during cell reprogramming on surfaces coated (**a** with or **b**) without vitronectin (VN). PS+VN is the positive control. White arrows indicate differentiated cells within hiPSC colonies, while black arrows indicate fully reprogrammed hiPSC colonies with a defined cell boundary.

Cell reprogramming of human fibroblasts into hiPSCs using episomal vectors in a feeder-free system was performed on different surfaces for 28 days, and the morphology and quality of the hiPSC colonies was analysed by bright-field microscopy. Figure 4 illustrates the changes in morphology of cells during reprogramming. Under conventional feeder free cell reprogramming conditions (PS+VN; vitronectin-coated non-tissue culture treated polystyrene), small colonies can be observed on the surface after 16 days (Fig. 4a). After 24 days, colonies resembling typical human embryonic stem cell (hESC) morphology, with a defined colony boundary (back arrows) and high nucleus to cytoplasmic ratio, were found on PS+VN as well as glass+VN, 2SiPM+VN, and 5SiPM+VN surfaces with various sizes. Notably, the colony size on 5SiPM+VN was much smaller compared with other surfaces. By day 28, we observed large undifferentiated colonies forming on 2SiPM+VN while some parts of the colonies started to differentiate on the control surfaces (white arrows; Fig. 4a).

During reprogramming without a VN coating, as expected we observed poor colony formation on PS with noticeable cell detachment at a late time point (day 28, Fig. 4b). Similarly, on the flat glass control we observed poor colony formation with most colonies containing differentiated cells (white arrows) and only a limited number of colonies having the expected hESC morphology. This is consistent with a previous report that the ECM protein coating, i.e. VN in this study, is important for cell reprogramming on PS^23^. In contrast, we observed formation of hiPSC colonies on both 2SiPM and 5SiPM even without the VN coating from day 24 onwards, with hiPSC colonies being noticeably smaller on 5SiPM (black arrows). These interesting results suggest that BCCs can be used as a replacement for substrates with adsorbed VN and are promising at supporting generation of hiPSC colonies in feeder free reprogramming conditions.

**Figure 5:**
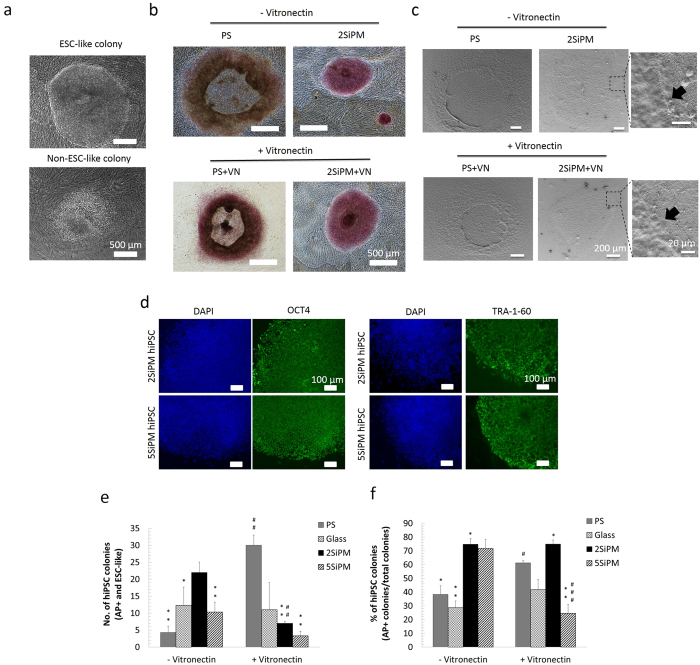
Assessment of cell reprogramming on BCCs. (**a**) hESC-like colony morphology of fully reprogrammed hiPSC colonies (upper image) and partially reprogrammed cells, i.e. a non-hiPSC colony (lower image) under a standard reprogramming protocol. (**b**) Alkaline phosphatase (AP) staining of hiPSC colonies. Fully reprogrammed hiPSCs were found on 2SiPM while differentiated iPSC colonies were found on PS with and without a VN coating. (**c**) SEM images show the colony-surface interactions in detail, in particular the edge of the colonies and the particles underneath. (**d**) Immunocytochemistry of pluripotent markers OCT4 (left) and TRA-1-60 (right) in a clonal hiPSC line derived from culture on the 2SiPM or 5SiPM surfaces. (**e**) Quantification of the number of fully reprogrammed hiPSC colonies, and (**f**) the percentage of fully reprogrammed colonies (hiPSC colonies/total colonies). Noted that hiPSC colonies are defined as AP positive with a hESC-like morphology. Value = mean ± SEM (*n* = 3). *vs. positive control, i.e. PS+VN, and ^#^vs. the same surface without a vitronectin (VN) coating at the same day. One, two, and three symbols indicate *p* < 0.05, 0.01, and 0.001, respectively.

To quantify the cellular reprogramming efficiency of the various surfaces tested, we performed alkaline phosphatase (AP) staining after 28 days of reprogramming and counted the number of hiPSC colonies based on hESC morphology and positive AP expression (Fig. 5). We have shown previously that the criteria for using AP expression coupled with hESC morphology represents a reliable method to quantify reprogramming efficiency, with similar accuracy to quantification based on TRA-1-60 expression (Supplementary Fig. 1)^20^. Figure 5a shows a representative picture of a fully reprogrammed hiPSC colony with hESC morphology and a non-hiPSC colony containing differentiated cells. Figure 5b shows representative pictures of AP expression in colonies observed on PS and 2SiPM. Noticeably, the 2SiPM surface yielded fully reprogrammed hiPSC colonies with high AP expression and hESC morphology, compared to AP negative colonies on the PS surface without a VN coating. Figure 5c shows details of the colony-surface interactions. From the high magnification images, the black arrows show the boundary of colony and the particle substrate surface.

Clonal hiPSC cell lines were isolated from reprogrammed colonies on2SiPM or 5SiPM substrates and showed positive expression of pluripotent markers, OCT4 and TRA-1-60 (Fig. 5d), further supporting the quality of the hiPSCs generated.

The positive control of PS with a vitronectin coating (PS+VN) yields the highest number of hiPSC colonies (30 ± 3 colonies), followed by 2SiPM (no VN) (22 ± 3 colonies) (Fig. 5e). The 5SiPM (no VN) reprogramming experiment yields 10 ± 3 colonies whereas the negative control PS (no VN) yields the least number of hiPSC colonies (4 ± 2 colonies). These results confirm that in the absence of a vitronectin coating both 2SiPM and 5SiPM represent an improvement to using PS for reprogramming. We also compared the effect of a VN coating on the various surfaces. Interestingly, although VN dramatically improved the number of hiPSC colonies on the PS surface (from 4 ± 2 to 30 ± 3), it seems to have an inhibitory effect on reprogramming on 2SiPM (from 22 ± 3 to 7 ± 1 colonies) and 5SiPM surfaces (from 10 ± 3 to 3 ± 1 colonies). On the other hand, we observed no significant effect of VN on the glass surface (Glass: 12 ± 5 colonies; Glass+VN: 11 ± 8 colonies).

Next, we quantified the number of partially reprogrammed colonies under different conditions, defined by the absence of AP expression and/or lack of hESC morphology (Supplementary Fig. 4). This allowed us to assess the proportion of fully reprogrammed hiPSC colonies out of all colonies that arise (Fig. 5f). Under conventional reprogramming conditions on the PS+VN surface, we typically observed ~61% of fully reprogrammed hiPSC colonies in the culture. In comparison, interestingly the 2SiPM BCC surface (with or without VN coating) significantly improved the proportion of fully reprogrammed hiPSC colonies to ~75% (Fig. 5f). Our results also demonstrate a high percentage of hiPSC colonies at ~72% supported on the 5SiPM BCC. In contrast, as expected under negative control conditions (PS and glass) we observed only ~28–38% of fully reprogrammed hiPSC colonies in the culture. Together, here we have demonstrated that the BCC surfaces improve the proportion of *bona fide* hiPSC colonies during reprogramming compared to conventional conditions, which has the potential to facilitate the isolation of hiPSC colonies for downstream applications.

## Discussion

Topography-induced cell behavioural changes including morphology, proliferation, and differentiation have been widely reported and the responses can be related to the size and geometry of the features that have been fabricated^24^,^25^,^26^. The organization of cell cytoskeletons, cell morphology, and focal adhesions are influenced by different surface topographies such as porosity, pillars, and grooves^27^,^28^,^29^,^30^,^31^. For example, on grooved patterns most mammalian cells including stem cells will elongate and align in parallel with the direction of the grooves^19^,^24^,^26^,^29^. This topography-induced alignment can improve the differentiation of mesenchymal stem cells (MSCs) into specific cell types, such as neurons^32^ and cardiomyocytes^33^, as well as facilitate direct reprogramming to these specific lineages^14^,^15^. Many studies have been dedicated to exploring the underlying biomolecular mechanisms of these topography-induced cellular responses. One remarkable report studying osteogenesis of MSCs using nanopitted surfaces suggested that the symmetry of the specific topography can trigger specific cell signalling and in turn improve osteogenic differentiation of hMSCs via pathways different to chemical induction^34^. In a subsequent study, another nanopitted surface with square arrangements was used to maintain the multipotency of MSCs^35^. The symmetry of these nanopitted surfaces plays a key role in directing hMSC behaviour. These studies support the idea that cellular behaviour can be modulated using a specific surface topography and/or chemistry arrangement alone. A desired cellular response can also be achieved using a combination of specific surface properties and effective growth factors present in the culture medium.

Generation of pluripotent stem cells from somatic cells has captured the imagination of scientists in the last decade. There are several techniques to generate pluripotent stem cells (PSCs) from somatic cells including somatic cell nuclear transfer (SCNT)^36^, cell reprogramming via cell fusion^37^, cell extracts^38^, or defined transcription factors^39^. The first derivation of hiPSCs was reported in 2007 by two independent groups. They used four transcription factors (OSMK or OSNL) to reprogram fibroblasts, providing a relatively easier approach that is more defined compared with previous methods^2^,^3^. With continued advances of technology, integration-free and feeder-free cell culture systems have been developed, which is one step closer to the clinic and is achievable in standard laboratories^20^. One study reported that surface topography may be able to replace the effects of small-molecule epigenetic modifiers and significantly improve reprogramming efficiency^13^. Our recent study showed that human MSCs formed clumps on BCCs^17^. In this study, we further demonstrate that specific surface properties may be able to replace the ECM protein coating by changing the cytoskeleton and possibly the way the protein adsorbs to the surface. This strong evidence suggests that optimal cell-surface interactions can improve the process of cell reprogramming, which will benefit stem cell research.

In the present study, cell reprogramming of human fibroblasts into hiPSCs was explored using a new family of cell culture substrate called BCCs. More specifically, two BCCs with highly ordered surface topography were selected from our library^17^,^21^. Due to the simplicity of the process, this fabrication process has the potential for large-scale manufacturing. These two selected BCCs have highly ordered surface topographies (i.e. hexagonal close-packed arrangement) and heterogeneous chemistries (i.e. Si and PMMA), which can be used for further selective chemical modification such as silanization of the Si particles. Modified BCCs may further change cellular responses in different way. BCC substrates can be manufactured easily in any size ranging from 0.19 cm^2^–7 cm^2^ with submicron features that are difficult to prepare using alternative methods. Random to semi-ordered surface topographies can be readily fabricated by changing the ratio and surface properties of colloidal particles used for self-assembly. Importantly, there is no report using BCCs or similar structures for cell reprogramming.

The two significant outcomes of this study are: 1) an improvement to the proportion of the fully reprogrammed hiPSC colonies using non-planar surfaces (i.e. BCCs); and 2) demonstration that BCCs are a promising alternative to replace the VN coating. Improving the proportion of fully reprogrammed hiPSCs will facilitate the establishment of clonal hiPSC cell lines. Using BCCs as a novel surface for reprogramming, we show here that 2SiPM allows for cell reprogramming with a high proportion of fully reprogrammed colonies (~75%). This effect is independent on VN coating, supporting the use of 2SiPM as an alternative for the VN layer used in cell reprogramming. While the survey spectra of XPS showed a different amount of VN, high resolution spectra showed a different VN structure between PS and 2SiPM. This may affect initial fibroblast adhesion (cell size and elongation), in turn influencing long term cellular response during cell reprogramming. It is also possible that the complex surface properties of BCCs induce different ECM protein synthesis in the cultured cells or influence protein deposition from the medium. We also noticed that 2SiPM contains specific chemical structure at 289 eV while the surfaces coated with VN there is a specific chemical structure at 288 eV. These chemical structures may be important for cell reprogramming with or without vitronectin coating. The precise nature of the protein adsorption on the BCCs will be investigated in the future.

Another BCC surface, the 5SiPM, with similar wettability and elemental composition can also support reprogramming with a high proportion of fully reprogrammed hiPSC colonies. However, its effect seems to decrease when combined with a VN coating. It is unclear why the VN coating has a negative effect on cell reprogramming using the 5SiPM. The XPS results revealed that the 5SiPM adsorbed the lowest levels of VN and this could be the reason why VN was in a less active state. In addition, the correct 3D structure of proteins is essential to their function^40^ as failure to fold into native structure generally produces inactive proteins or toxic functionality^40^. As we show here, the complex surface properties of BCCs can modulate cell adhesion, density and proliferation of fibroblasts, which could ultimately influence the generation of hiPSCs. From the results of this study, we speculate that an optimal cell size, shape and proliferation can be modulated by synergistic biochemical and biophysical cues and in turn improve the process of cellular reprogramming.

It is important to note that different reprogramming methods may generate a different outcome of cell reprogramming, thus further studies to test other feeder-free reprogramming methods using BCCs will be needed. Also, as different somatic cells are reported to yield different reprogramming efficiencies^41^, future research that utilizes other cell types for reprogramming on BCCs will address variations in reprogramming efficiencies.

Several studies have reported improvements to the efficiency of cell reprogramming by alteration of reprogramming factors, surface matrix, media composition, and starting cell types^42^. However, the issue with partially reprogrammed colonies during hiPSC generation is rarely discussed, with limited advance in the effort to minimize partially reprogrammed cells and promote fully reprogrammed cells to propagate. This study reports on the development of a feeder-free system for generation of a high proportion of fully reprogrammed hiPSCs using a specific, well defined surface topography. Although we did not observe any improvement in overall reprogramming efficiency compared to conventional reprogramming using a vitronectin coated PS surface, the results are extremely interesting since they suggest the BCCs exert a negative selection on cells that fail to undergo full reprogramming, resulting in significant increases in the percentage of fully reprogrammed colonies in culture. This proof-of-concept study demonstrates that specific surface properties (topography and chemistry) have the potential to replace extracellular matrix coatings, influence cell growth and in turn improve cellular reprogramming processes to generate more fully reprogrammed hiPSCs. This reprogramming system will help simplify the isolation of hiPSC colonies to establish clonal cell lines and facilitate the application of hiPSC in regenerative medicine and disease modelling.

## Additional Information

**How to cite this article**: Wang, P.-Y. *et al*. Binary colloidal crystals (BCCs) as a feeder-free system to generate human induced pluripotent stem cells (hiPSCs). *Sci. Rep*. **6**, 36845; doi: 10.1038/srep36845 (2016).

**Publisher’s note**: Springer Nature remains neutral with regard to jurisdictional claims in published maps and institutional affiliations.

## Supplementary Material

Supplementary Information
