# Supplementary material for: Binary colloidal crystals (BCCs) as a feeder-free system to generate human induced pluripotent stem cells (hiPSCs)
Source: Sci Rep. 2016 Nov 11;6:36845. doi: 10.1038/srep36845 (PMC5104981; doi:10.1038/srep36845)
Supplement: Supplementary Information [file srep36845-s1.pdf]

Binary colloidal crystals (BCCs) as a feeder-free system to generate human induced pluripotent stem cells (hiPSCs)

Peng-Yuan Wang[1-4*], Sandy Shen-Chi Hung[5], Helmut Thissen[4], Peter Kingshott[1], and Raymond Ching-Bong Wong[5]

1. Department of Chemistry and Biotechnology, Faculty of Science, Engineering and Technology, Swinburne University of Technology, Hawthorn, Victoria 3122, Australia
2. Graduate Institute of Nanomedicine and Medical Engineering, College of Biomedical Engineering, Taipei Medical University, Taipei 110, Taiwan
3. Department of Anatomy and Neuroscience, Florey Neuroscience and Mental Health Institute, The University of Melbourne, Victoria 3000, Australia
4. CSIRO Manufacturing, Bayview Avenue, Clayton, 3168 Victoria, Australia
5. Centre for Eye Research Australia & Ophthalmology, Department of Surgery, the University of Melbourne, Victoria 3002, Australia

Supporting Information

Table S1. XPS elemental analysis on surfaces used in this study (elemental concentration in %)

	C	N	O	Si	Na	Cl	P	Zn
PS	98.6		1.4					
	99.1		0.9					
	99.0		1.0					
	99.0		1.0					
	99.2		0.8					
	99.3		0.8					
	99.0		1.0					
	98.6		1.4					
	98.8		1.2					
	99.3		0.7					
	99.0		1.0					
Glass	11.1		64.5	20.1	3.9			0.5
	10.8		64.6	20.5	3.6			0.5
	11.1		64.2	19.8	4.3			0.6
	10.9		65.3	20.1	3.2			0.5
	10.9		68.6	19.4	0.6			0.5
	12.1		63.7	19.8	3.9			0.5
2PM	59.7		36.5	3.8				
	50.8		40.0	9.1				
	51.1		39.6	9.3				
	58.0		36.7	5.3				
	55.4		38.7	5.9				
	56.3		38.0	5.8				
5PM	59.3		33.9	6.9				
	58.2		34.5	7.4				
	58.6		34.6	6.9				
	60.8		33.4	5.8				
	61.0		33.1	5.9				
	59.8		33.7	6.5				
PS+VN	43.3	7.9	26.0		9.9	9.9	2.9	
	40.6	8.6	27.9		10.2	9.7	3.1	
	40.4	8.2	27.5		10.5	10.3	3.1	
	43.7	8.0	24.7		10.1	11.0	2.5	
	32.9	7.4	32.8		11.0	11.3	4.6	
	29.4	5.4	32.7		14.5	13.5	4.5	
Glass+VN	20.8	4.1	52.7	4.9	10.7	4.2	2.6	
	23.4	4.5	51.1	5.1	9.6	4.6	1.8	
	43.1	2.0	37.6	2.0	7.6	7.0	0.8	
	25.0	4.1	45.4	3.7	11.5	8.7	1.7	
	24.0	5.5	44.6	3.7	11.2	8.5	2.5	
2PM+VN	39.9	3.8	27.3	0.4	13.9	12.0	2.7	
	28.2	3.4	52.3	7.2	5.1	2.6	1.1	
	33.6	5.8	45.1	6.1	5.2	3.8	0.4	
	41.3	2.9	35.1	2.0	9.5	6.9	2.3	
	40.6	2.8	32.2	1.2	11.2	9.1	2.9	
	24.3	3.9	54.1	6.9	6.4	2.8	1.7	
5PM+VN	54.5	2.3	33.2	2.4	3.8	3.3	0.6	
	51.3	2.2	33.8	2.6	5.0	4.4	0.7	
	51.9	3.0	32.3	2.1	5.7	4.6	0.4	
	53.8	3.5	34.5	2.9	2.7	2.3	0.4	
	47.2	1.5	28.7	0.8	10.9	10.2	0.7	
	45.0	2.3	30.3	1.4	10.9	9.5	0.6	

Figure S1.

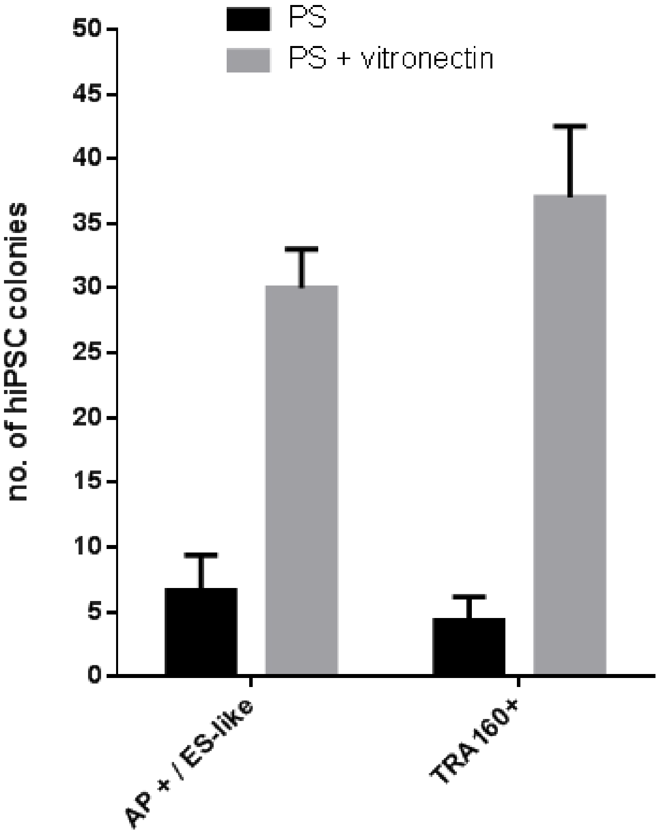

Figure S1. Quantification of hiPSC generation as assessed by the number of TRA-1-60 positive colonies or AP^+ and hESC-like colonies. At day 28 post-reprogramming, the hiPSC cells were immunostained live with TRA-1-60 and quantified, followed by AP staining and colonies counting. Value = mean ± SEM (*n* = 3). Note that data for TRA-1-60^+ colony numbers for PS + vitronectin condition was published previously.[1]

Figure S2.

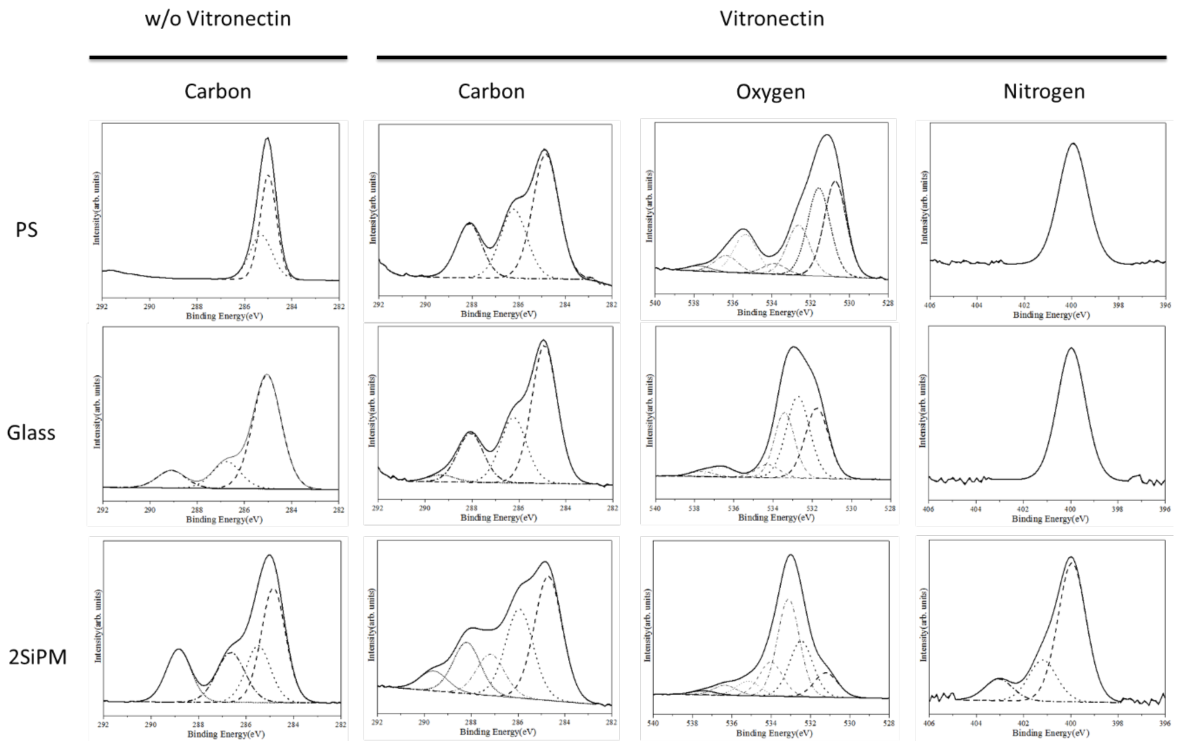

Figure S2. Representative high resolution spectra before and after vitronectin coating on the various surfaces. Spectra shown are carbon, oxygen, and nitrogen. Curve fitting was performed using CasaXPS software with a residual STD < 1. The first column shows the original surface chemistry and the right three columns show the surface chemistry after vitronectin coating for 1h.

Figure S3a.

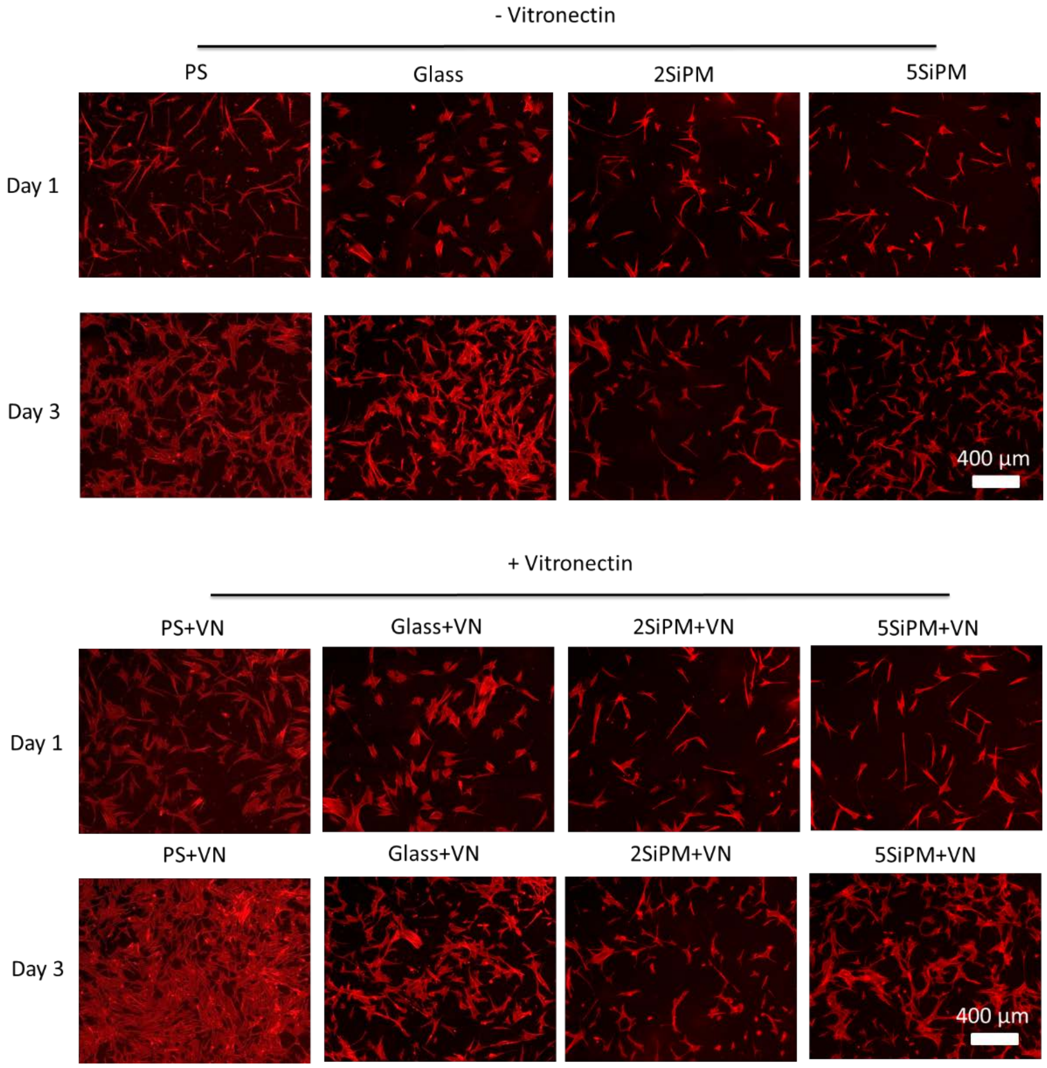
- Vitronectin
PS
Glass
2SiPM
5SiPM
Day 1
Day 3
400 µm
+ Vitronectin
PS+VN
Glass+VN
2SiPM+VN
5SiPM+VN
Day 1
PS+VN
Glass+VN
2SiPM+VN
5SiPM+VN
Day 3
400 µm

Figure S3b.

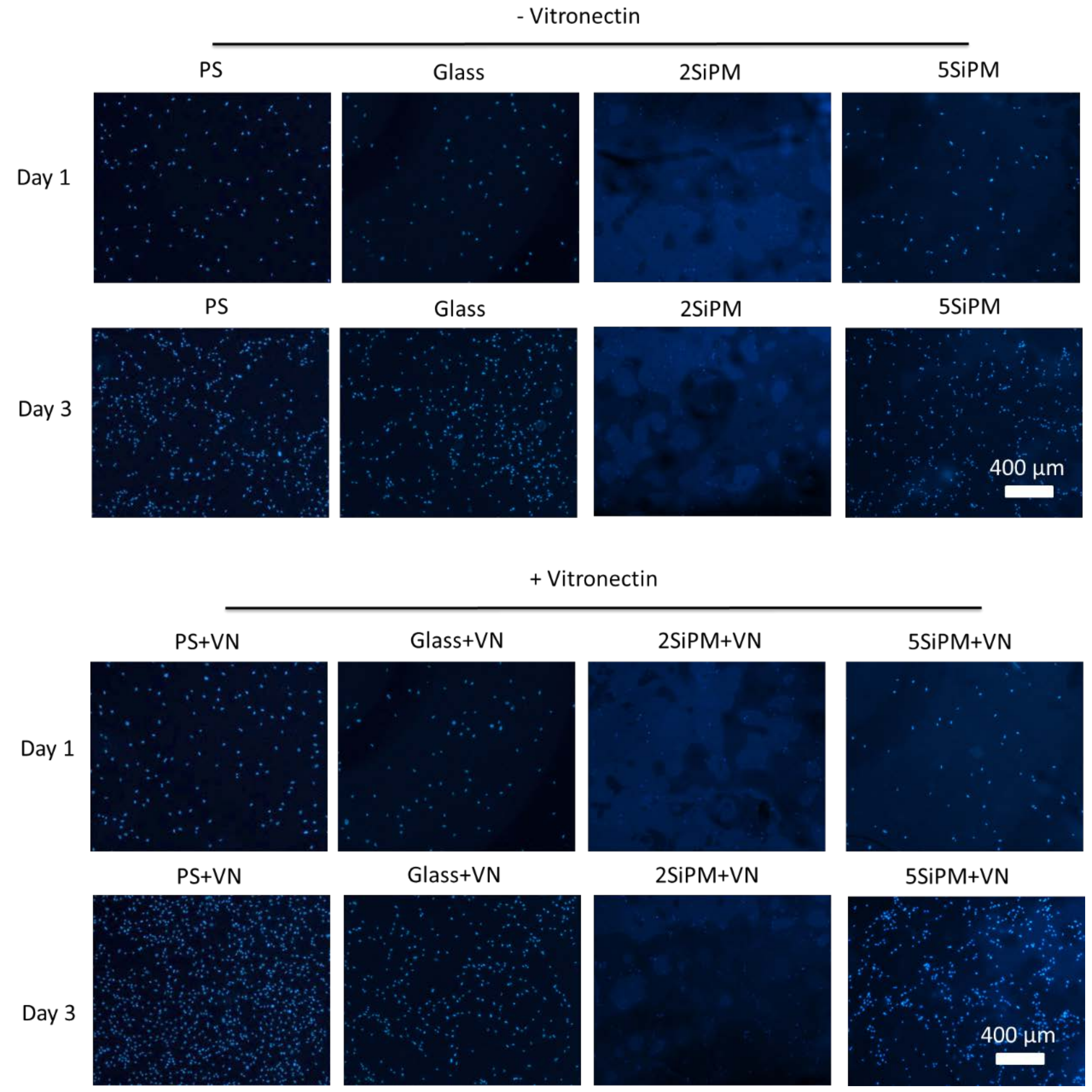

Figure S3. Representative images (4x) of fibroblasts cultured on different surfaces immunostained with (a) F-actin and (b) DAPI counterstain. Note that the 2SiPM surface exhibited some autofluorescence background due to the silica particles present.

Figure S4.

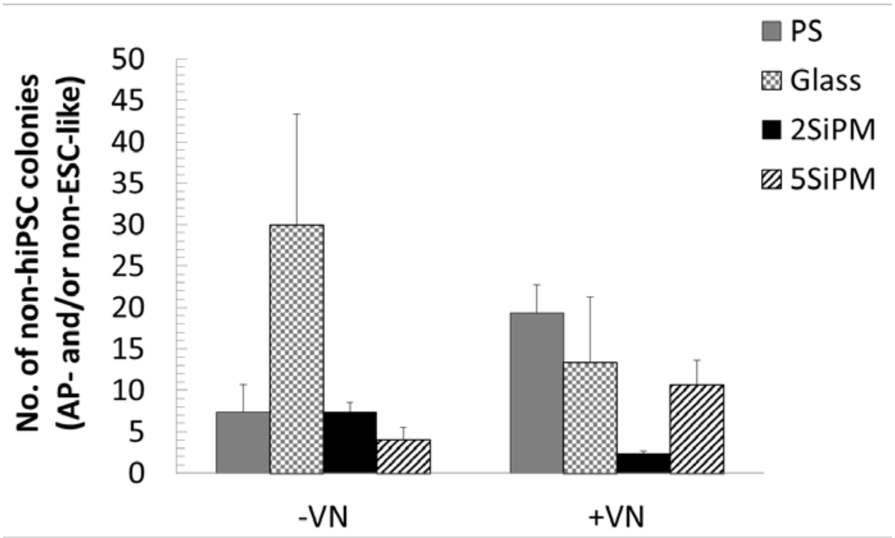

Figure S4. Quantification of partially reprogrammed colonies (AP negative and/or exhibit non-ESC morphology) after 28 days of cell reprogramming on various surfaces. Value = mean ± SEM (*n* = 3).

References

1. Hung SS, *et al.* Study of mitochondrial respiratory defects on reprogramming to human induced pluripotent stem cells. *Aging (Albany NY)* **8**, 945-957 (2016).
